# An Adhesion Improvement of Low-Density Polyethylene to Aluminum through Modification with Functionalized Polymers

**DOI:** 10.3390/polym15040916

**Published:** 2023-02-11

**Authors:** Mohamed Nassr, Igor Krupa, Mabrouk Ouederni, Senthil Kumar Krishnamoorthy, Anton Popelka

**Affiliations:** 1 Center for Advanced Materials, Qatar University, Doha P.O. Box 2713, Qatar; 2 Product Development & Innovation, Qatar Petrochemical Company (QAPCO), Doha P.O. Box 756, Qatar

**Keywords:** low-density polyethylene, bulk modification, maleated polyethylene, adhesion, wettability

## Abstract

An interfacial adhesion improvement between low-density polyethylene (LDPE) and aluminum (Al) foil is an important challenge in designing multilayered packaging (TetraPak packaging type) due to insufficient inherent adhesion between both untreated materials. Therefore, extra adhesive layers are often used. The hydrophobic character of LDPE is responsible for poor adhesion to Al and can result in delamination. This study deals with the comparative study of the bulk modification of LDPE with various commercially available adhesive promoters with different chemical compositions to increase LDPE’s adhesive characteristics and ensure good adhesion in LDPE/Al laminates. A copolymer of ethylene and methacrylic acid; a terpolymer of ethylene, maleic anhydride, and acrylic ester; or maleated polyethylene (PE) were used as adhesive promoters, and their effect on adhesion improvement of LDPE to Al was investigated. The best adhesion improvement was observed in LDPE-modified samples with maleated PE, while 0.1 wt.% additive content significantly increased peel resistance (from zero to 105 N/m). An additional increase in additive content (0.5 wt.%) in LDPE led to stronger adhesion forces than the cohesion forces in Al foil. Adding 0.5 wt.% of maleated PE into LDPE improved the LDPE/Al laminates’ adhesion and can be applied in multilayered lamination applications.

## 1. Introduction

A unique characteristic of LDPE is its low manufacturing cost and ability to be fabricated into a wide range of products [1,2,3]. LDPE is the most commonly used plastic in the world because it has thermal stability and chemical inertness [4,5,6]. In addition, LDPE possesses several excellent properties that make it a perfect candidate for specific applications. LDPE is considered a safe and moisture-resistant [7] material with low toxicity [8,9], reactivity [10], flammability [11], and low melting point [12], which makes it ideal for products intended for use in food packaging [13,14]. LDPE is commonly used in food packaging in combination with aluminum foil (TetraPak). Heat-sealing qualities are provided by LDPE, whereas barrier and mechanical properties are provided by Al [15]. Metals have higher surface free energy than polymers, and therefore, the adhesion strength of a metal adhesive system is higher than a polymer adhesive system. Some methods exist for an enhancement of the adhesion strength in a polymer adhesive system, for example, etching by acid [16], plasma treatment [17], or ozone/UV treatment [18]; however, these methods are not effective enough or permanent [19].

The strong interfacial adhesion between the LDPE and Al foil can be achieved by adding additives to LDPE responsible for increasing hydrophilicity, resulting in better wettability [20,21]. Interfacial adhesion happens when two different materials are bonded together physically. The surface of Al foil is hydrophilic, while the LDPE surface is hydrophobic; therefore the interfacial adhesion can be increased by increasing the hydrophilicity of LDPE by adding polar groups [22,23,24,25,26]. Adhesion knowledge is crucial in various industries, including packaging, automobiles, aeronautics, aerospace, electronics, and sports [27]. Surface chemistry, physics, polymer chemistry, rheology, mechanics, physics, fracture analysis, and many other disciplines are all involved in adhesion [28]. Coatings, polymer mixtures, paints, multilayered sandwiches, adhesive junctions, and composite materials all exhibit two different types of adhesion: intrinsic adhesion and measurable adhesion. The first refers to the immediate molecular attraction forces in substrates and adhesives. The second is generated from the strength evaluation of the adhesive junction. Intrinsic adhesion forces across the interface are necessary for adhesion formation [29,30,31]. The four main adhesion theories are adsorption, electrostatic attraction, diffusion, and mechanical interlocking. According to the adsorption principle, forces cause mobile phase macromolecules such as ink, glue, and printing to adsorb into the substrate (in the range from dispersion to chemical bonds) [32]. According to electrostatic theory, when two materials with distinct band structures come together at an interface, a charge transfer binds them together. Only incompatible materials can be used in this method, such as polymer adhesion to a metallic surface [33]. The diffusion theory examines how macromolecules move from the mobile phase into the substrate when an interface is removed. The final hypothesis illustrates the interlocking action as the mobile phase flows into the substrate surface defects (mechanical interlocking) [34]. When a polymer in the molten state is poured onto the surface of the metal, the polymer can penetrate into the rough structures of the metal and form mechanical interlocking after curing [35]. In the earliest adhesion theory, the substrate’s porosity and roughness are sufficient elements since the adhesive’s wettability is sufficient, whereas non-wetted areas lead to failures [32].

A weak boundary model represents a unique sort of adhesion. The contact between the adhesive and the substrate would not fail in this model, but the failure would be driven by creating a weak boundary layer [36]. As a result, in order to achieve good adhesive qualities, the weak border layer should be removed. Except for Al, which has a coherent oxide layer, this problem can occur in metals with a scaly oxide layer that causes failure at the boundary [37]. The weak adhesion interface is also caused by a low molecular weight component in PE, which can be eliminated by surface preparation [38]. In order to achieve enhanced interfacial adhesion, both materials must possess the same properties. A hydrophobicity equal between the two polymers can create a strong bond between the two materials. With this property, more excellent dimensional stability can be achieved [39,40].

Adhesion enhancers can be used as additives or pretreatments in general. The promoter is used as a prepared primer or solution in a suitable solvent or solvent combination [41]. The significant adhesion promoters applied as coatings are silanes with the overall structure R-Si(OR’)_3_, where R is an organofunctional group and R’ is a hydrolyzable group. Silanes contain polar silanol and organofunctional groups that can interact with polymers. An example of commonly used adhesion promoters applied as additives are ethylene/acid copolymer ionomers containing (meth)acrylic acid and dicarboxylic acid monomers. These copolymers can be melt-processed with polymers to improve adhesion characteristics having adhesive and polymer blend compatibilizing characteristics [42]. Maleic anhydride is another commonly used adhesion additive promoter to improve adhesion between polymer and metal surfaces [43,44,45]. The maleic anhydride, which is grafted to thermoplastics, such as polyethylene or polypropylene, can improve wettability and interfacial adhesion [46,47]. In the early adhesion stages, maleic anhydride can be hydrolyzed to maleic acid on native Al oxide, while one of its acid groups is a chelate bridged to two Al atoms. A monodentate bond is then identified between the acid group (carbonyl oxygen) and an Al [44].

In this study, the bulk modification of LDPE with various commercially available adhesive promoters with different chemical natures was used to increase LDPE’s adhesive characteristics and ensure good adhesive joints in LDPE/Al laminates applicable in the packaging industry and potentially replace commonly used extra adhesive layers. A copolymer of ethylene and methacrylic acid [48]; a terpolymer of ethylene, maleic anhydride, and acrylic ester [49]; or maleated PE [50] were used as adhesive promoters, and their effect on adhesive characteristics of LDPE was analyzed.

## 2. Materials and Methods

### 2.1. Materials

LDPE (LDPE EC-02 type) pellets for the preparation of laminate with Al were kindly provided by Qatar Petrochemical Company (QAPCO, Doha, Qatar). The granular LDPE was transformed into thin sheets by a mounting hot press machine (Carver 3895, Wabash, IN, USA) for 3 min at 160 °C with subsequent cooling to RT with a water medium. Table 1 summarizes the fundamental physical properties of this LDPE grade. Furthermore, the following materials were used. Acetone (Research-Lab, Mumbai, India, 99.5%) was applied to remove contaminants/impurities from the Al and LDPE surfaces before the lamination process. Al foil (Freshwrapp, 0.4 mm thick, Hindalco Industries Ltd., Mumbai, India) was used for the preparation of the LDPE/Al laminates.

NUCREL™ 960 (Dow packaging, a copolymer of ethylene and 15 wt.% of methacrylic acid, NUCREL™ 1202 (copolymer of ethylene and 11.5 wt.% of methacrylic acid, NUCREL™ 599 (copolymer of ethylene and 10 wt.% of methacrylic acid, made with nominally methacrylic acid), OREVAC^®^ 18302N (maleic anhydride modified LDPE), LOTADER^®^ 3410 (random terpolymer of ethylene, maleic anhydride, and acrylic ester polymerized by autoclave process at high pressure), poly(ethylene-co-methacrylic acid) (PEMA) (Sigma-Aldrich, St. Louis, MO, USA) were used as adhesive promoters. Ultra-pure water (purity > 99%, prepared by a water purification system, Direct-Q^®^, Molsheim, France), ethylene glycol (purity 98 percent, FLUKA, Belgium), and formamide (purity > 98%, FLUKA, Morris Plains, NJ, USA) were used as testing liquids with different surface tensions to measure the contact angle and to determine a surface free energy and its polar and dispersive components.

### 2.2. Samples Preparation

Using a Plastrophotograph EC & Mixer 50EHT (Brabender, Duisburg, Germany) internal mixer at 30 revolutions per minute and 160 °C, the LDPE was blended with various additives having different masses. Then, thin, smooth films made of LDPE granules were fabricated using a hydraulic mounting press machine (Carver 3895, Wabash, IN, USA). First, 10 g of LDPE granules were placed between 2 polyester sheets and between steel plates. Then, the prepared samples were placed between the hydraulic press machine platens. The temperature was 160 °C, and 1 ton of load was applied to obtain thin films, which were cooled down to RT after 2 min. The LDPE films’ thickness measured with a Vernier caliper was about 320 µm.

The mounting press machine was also used to make the LDPE/Al laminate, which followed almost the same steps as in the case of the preparation of LDPE sheets. The LDPE unmodified/modified films, firstly washed with acetone, were directly placed on the glossy Al foil side and progressively heated to 160 °C, then, 2 tons of compression load was applied for 2 min to the LDPE/Al laminate, and then cooled to RT.

### 2.3. Wettability Analysis

Wettability changes in the LDPE samples were measured using a contact angle measurement system OCA 35 (Dataphysics, Filderstadt, Germany) with an optical video-base system containing a high-resolution CCD camera. The conventional Owens–Wendt–Rabel–Kaelble method [51] was used to assess the total surface free energy, and its polar and dispersive components using three testing liquids: water, formamide, and ethylene glycol (varying surface tensions). Each testing liquid was dispensed in a droplet of about 3 µL, and the contact angle with the samples was measured. The contact angle was evaluated approximately after 3 s (thermodynamic equilibrium between the liquid and the sample interfaces). Contact angles and surface free energy were evaluated using SCA20 software, V4.4.1

### 2.4. Adhesion Investigation

For the study of adhesion in LDPE/Al coherent laminate, a 90° peel test was used. A peel tester LF-Plus (Lloyd Instruments, West Sussex, UK) was used for the adhesion measurements, and NEXYGENPlus software was used for determining peel resistance (peel force per sample width). Before starting the test, laminated LDPE/Al strips (2 cm × 8 cm) were stuck on a double-sided adhesive tape (3M 4910k, VHBTM). The peel resistance was measured using a 100 N load cell at a 90° angle, while the LDPE was delaminated from the Al surface at a 10 mm/min delaminating rate. The measurement time was set at 6 min to ensure that the peel resistance was obtained from a sufficiently large area following ASTM D6862. Five independent runs were performed to obtain the peel resistance average values with standard deviations.

### 2.5. Mechanical Properties Investigation

A universal testing machine LF-Plus (Lloyd Instruments, West Sussex, UK) using a 1 kN load and strain stress of 10 mm/min was used to analyze the mechanical properties of LDPE samples at RT according to ASTM D638 using dog-bone specimens cut from 1 mm thick mounting hot-pressed slabs. The five independent specimens were analyzed from each sample to obtain the average value and standard deviation.

### 2.6. Surface Morphology Analysis

The topography/morphology and roughness of modified polymer surfaces relate to wettability and adhesion. Scanning electron microscopy (SEM) was used to focus on the surface morphology (2D) of modified samples, and the surface topography (3D) analyses were carried out using the atomic force microscopy AFM technique. The captured images indicated surface morphology/topography after the modification step and after peel tests to understand an adhesion mechanism. The information about the surface morphology of the prepared samples was obtained by SEM microscope Nova NanoSEM 450 (FEI, Hillsboro, OR, USA) using a detector of secondary electrons. The samples were coated with a Au layer (a few angstroms) for better resolution.

AFM was used to obtain detailed information about surface topography characteristics from small surface areas before and after peel tests. The measurements were performed using an AFM MFP-3D system (Asylum Research, Abingdon, Oxford, UK). This equipment contains a silicone AFM tip cantilever (Al reflex-coated Veeco model—OLTESPA, Tokyo, Olympus) with 2 N·m^−1^ and 70 kHz parameters, spring constant, and resonant frequency, respectively. All measurements were carried out using taping mode (non-contact) under ambient conditions in the air, and AFM images were obtained from a 5 × 5 µm^2^ surface area. This technique also provides information about surface roughness (Ra, a mean arithmetic height).

### 2.7. Chemical Composition Investigation

The changes in the chemical composition of the modified LDPE from the surface area were analyzed by Fourier transform infrared spectroscopy (FTIR) with an attenuated total reflectance accessory. An FTIR spectrometer Spectrum 400 (PerkinElmer, Waltham, MA, USA) allowed analyzing the samples in the mid-infrared (4000–500 cm^−1^) region to detect chemical composition changes in the modified samples. This technique provided qualitative data about the chemical composition from a small sample depth (microns) using ZnSe crystal. The FTIR spectra were obtained at a spectral resolution of 4 cm^−1^ and 8 scans using the absorbance mode.

## 3. Results

### 3.1. Adhesive Strength Measurements

**Figure 1:**
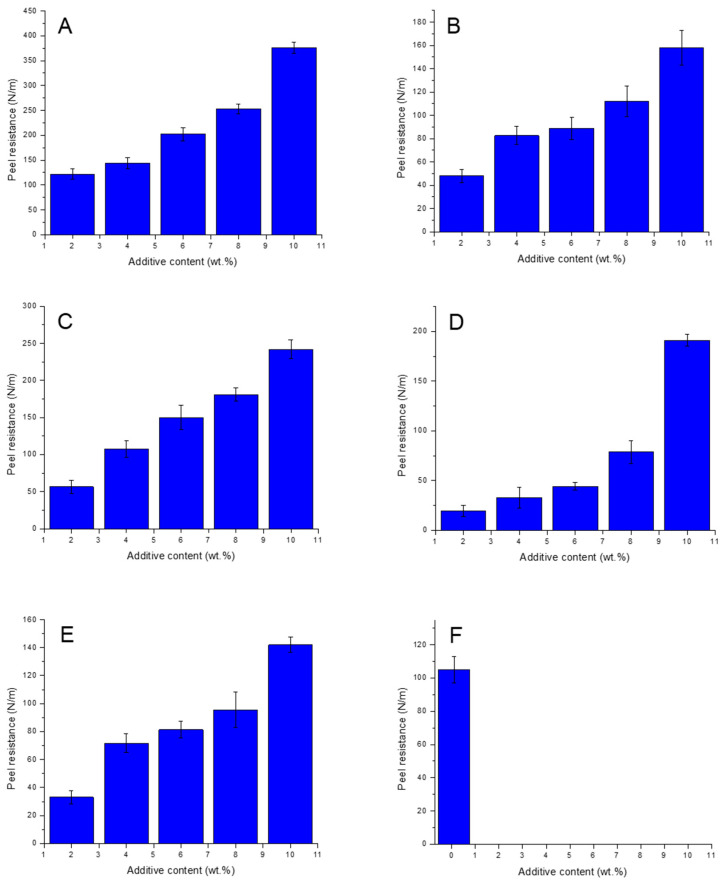
Peel resistance of LDPE/Al modified with (**A**) NUCREL 599, (**B**) NUCREL 960, (**C**) NUCREL 1202, (**D**) LOTADER, (**E**) PEMA, (**F**) OREVAC.

Due to the hydrophobic nature and low wettability of unmodified LDPE surfaces, the peel resistance of the LDPE/Al laminate was almost zero (not measurable), and LDPE was peeled out of Al even during manipulation. The peel resistance of modified LDPE/Al laminates increased as the additive concentration increased due to the increase in hydrophilic functional groups. As seen in Figure 1, all additives were responsible for improved peel resistance by increasing the concentration. The adhesion mechanism in polymer/metal laminate depends on different factors, such as mechanical interlocking, electrostatic attraction between the polymer and the metal substrates, or weak boundary layers in the region neighboring the laminate interface [52]. The best adhesion was observed for 10 wt.% for all used additives except maleated PE (OREVAC), while even very low content (0.1 wt.%) led to remarkable improvement in the peel resistance (105.0 N/m). An additional increase of maleated PE content (0.5 wt.%) in LDPE led to significant adhesion improvement, while Al foil was torn during measurement, so peel resistance could not be obtained. Based on these results, it can be concluded that adding 0.5 wt.% of maleated PE into LDPE improved the adhesion of LDPE-maleated PE/Al laminates satisfactorily, which can be applied in applications where strong adhesion is required.

### 3.2. Mechanical Properties Investigation

Mechanical properties of unmodified LDPE and LDPE modified with maleated PE (OREVAC) were analyzed by tensile tests. Obtained information about tensile strength and Young’s modulus is summarized in Table 2. Tensile strength and Young’s modulus of unmodified LDPE achieved values of 12.2 ± 0.9 MPa and 288.6 ± 18.6 MPa, respectively. A modification of LDPE with maleated PE led to negligible deterioration in mechanical properties due to a relatively low additive content and homogeneous bulk modification. Tensile strength and Young’s modulus of LDPE modified by 0.1 wt.% OREVAC dropped to 11.3 ± 1.4 MPa and 269.50 ± 21.5 MPa, respectively. An additional increase in additive content in the LDPE samples (0.5 wt.% OREVAC) resulted in a slight decrease in tensile strength (10.6 ± 0.5 MPa) and Young’s modulus (252.95 ± 7.5 MPa).

### 3.3. Surface Wettability Analysis

**Figure 2:**
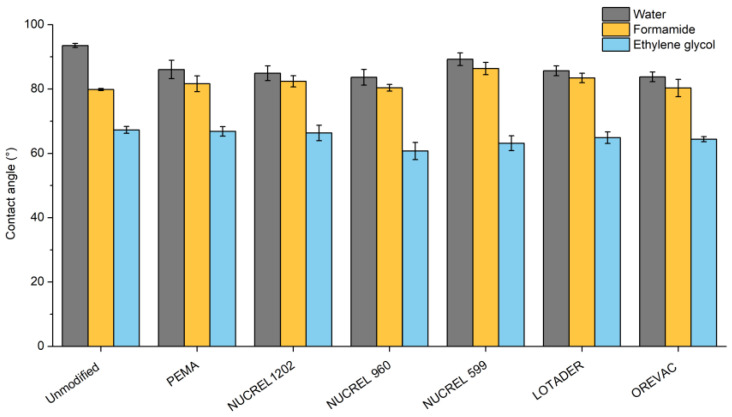
Contact angles of water, formamide, and ethylene glycol of LDPE samples.

Wettability is a liquid behavior that remains in contact with a solid substrate and is described by the contact angle. In this study, the surface free energy of the LDPE surfaces was assessed utilizing the contact angle measurements of unmodified and modified LDPE using three different testing liquids with variable surface tensions and polarity. Per the hydrophobic nature of LDPE, as demonstrated in Figure 2, the contact angles of unmodified LDPE attained comparatively high values (93.5°, 79.8°, and 67.3° for water, formamide, and ethylene glycol, respectively). The contact angle values, on the other hand, did not significantly change when LDPE and additives were combined, suggesting that the additives were mostly localized in the bulk of the LDPE matrix. Furthermore, by the attraction forces of additives present in bulk, polar additives may diffuse deeper beneath layers to achieve thermodynamic equilibrium.

**Figure 3:**
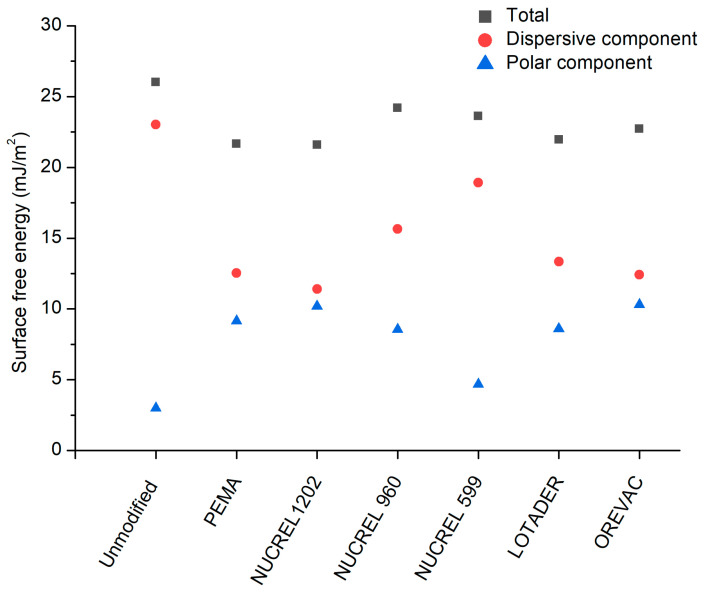
Surface free energy and its components of LDPE samples.

In order to examine the impact of adding additivities on the properties of solid–liquid interactions, the surface free energy was analyzed for unmodified and modified LDPE samples according to obtained contact angles, as shown in Figure 3. The contact angle data were used to calculate the surface free energy and its component values, and it was determined that there had been no appreciable change. Based on surface morphology and topography analysis, adding additivities did not change the surface properties significantly, as additives were homogeneously mixed with LDPE in bulk. However, polar component values increased after modification, indicating that polar functional groups were present on the material’s surface.

### 3.4. Chemical Composition Investigation

**Figure 4:**
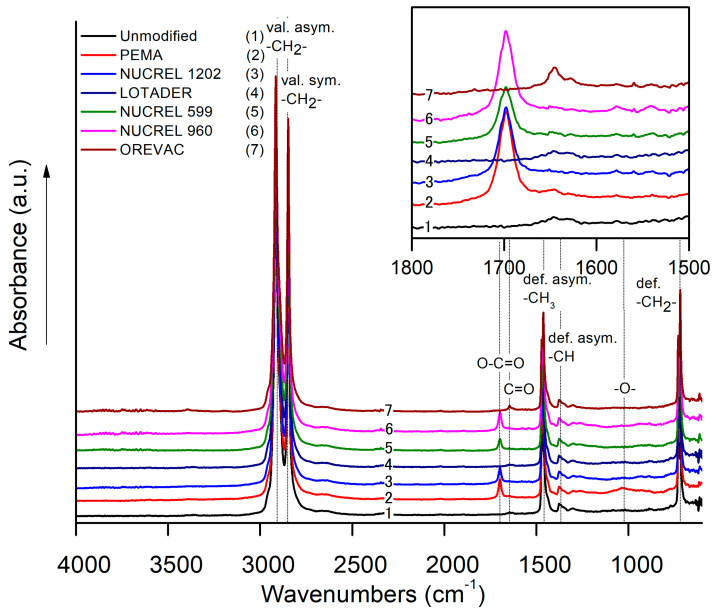
FTIR spectra of unmodified LDPE (1) and LDPE modified with PEMA (2), NUCREL 1202 (3), LOTADER (4), NUCREL 599 (5), NUCREL 960 (6), OREVAC (7).

FTIR was used to obtain information about the chemical composition on the surface of LDPE samples. The characteristic FTIR spectrum of the unmodified LDPE surface was associated with peaks representing non-polar LDPE groups shown in Figure 4; these peaks are CH_2_ asymmetric C-H stretch at 2917 cm^−1^, CH_2_ symmetric C-H stretch at 2852 cm^−1^, CH_3_ umbrella mode at 1377 cm^−1^, and CH_2_ rock at 718 cm^−1^ [53]. However, new peaks for the modified LDPE samples with additives appeared in the range of 1600 to 1750 cm^−1^, and these represent mainly the polar carbonyl functional groups C=O (1650 cm^−1^ at peak maximum) and C=O in the carboxylic group (1700 cm^−1^ at peak maximum) originating from the used additives. FTIR spectra analysis confirmed the presence of additives in the top and underneath layers of LDPE (~1.66 µm penetration depth for ZnSe crystal), which are crucial for forming adhesive joints with high adhesion characteristics.

### 3.5. Surface Morphology/Topography Analysis

**Figure 5:**
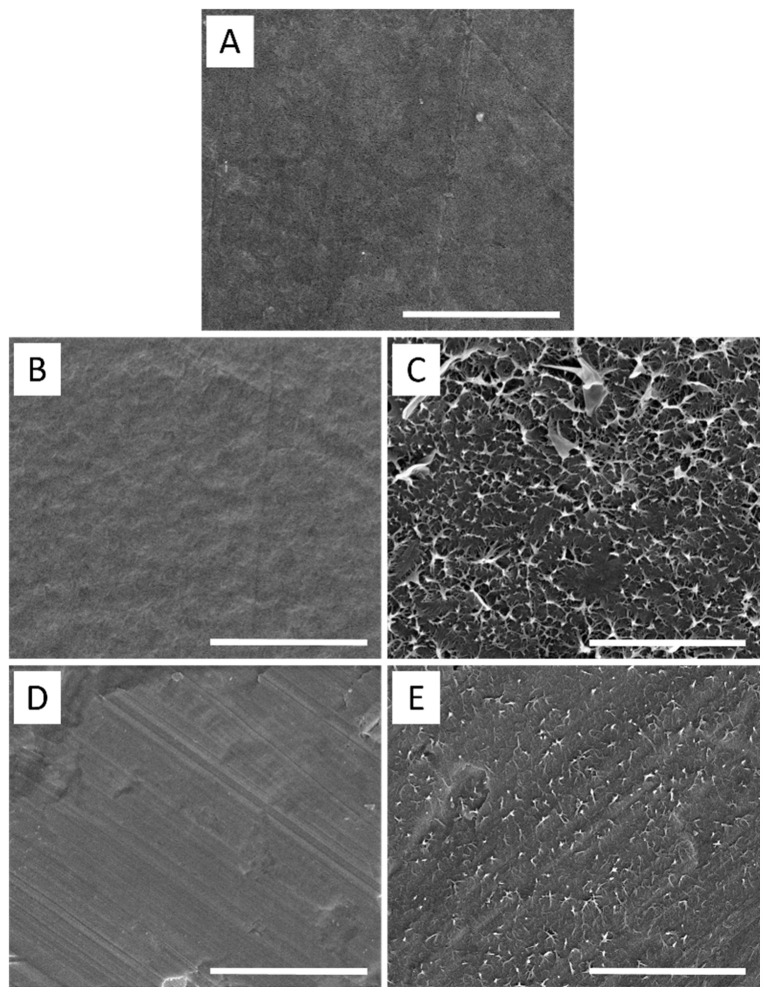
SEM images of (**A**) LDPE, (**B**) LDPE/Maleated PE (OREVAC), (**C**) LDPE/Maleated PE (OREVAC) after peel test, (**D**) Al, (**E**) Al after peel test; scale bare represents 5 µm.

Figure 5 shows 2D SEM images taken to investigate the change in the morphological structures of the LDPE samples and Al samples before and after peel tests. As shown in Figure 5A, surface of unmodified LDPE was relatively smooth, which can contribute to LDPE’s low wettability (surface free energy). Figure 5B represents the modified surface of LDPE, and it is seen that the addition of 0.1 wt.% of maleated PE did not change the surface roughness significantly, as the additive was homogeneously mixed with LDPE in bulk. However, according to Figure 5C, the surface roughness of the LDPE after peeling has increased after adding maleated PE with a percentage of 0.1 wt% due to the presence of the hydrophilic functional groups such as the carbonyl group and ether group according to maleic anhydride structure, which was protruded on the LDPE surface after a peel test. Moreover, the Al surface roughness also increased after the peeling test, which means the polar additives were attached to the polar Al surface, indicating a mechanical interlocking mechanism of adhesion.

**Figure 6:**
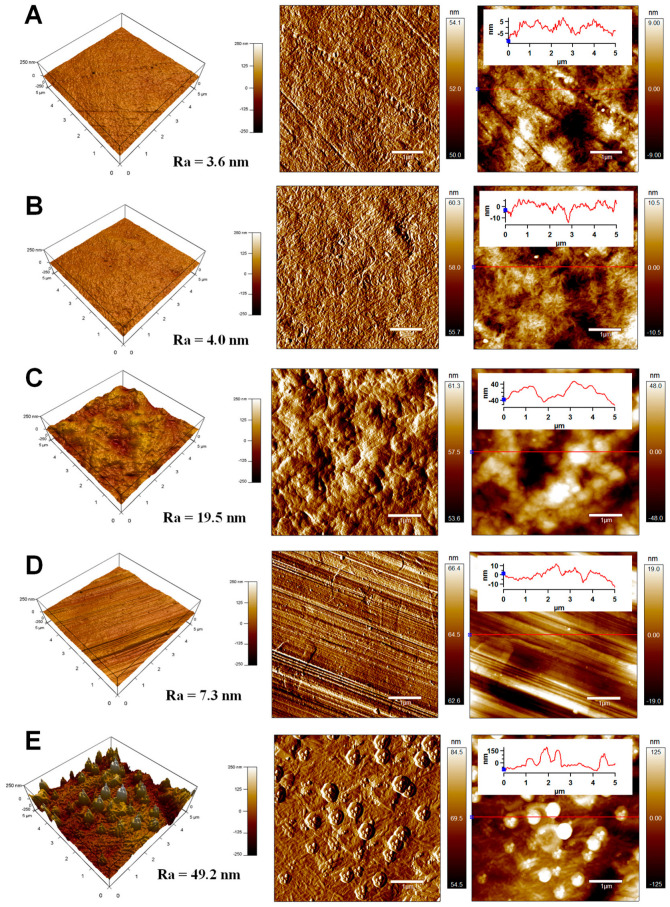
AFM images (3D height, amplitude, ZSensor with line profile) of (**A**) LDPE, (**B**) LDPE/maleated PE (OREVAC), (**C**) LDPE/maleated PE (OREVAC) after peel test, (**D**) Al, (**E**) Al after peel test.

The information about the surface morphology/topography of LDPE and Al samples from the 5 × 5 µm^2^ before and after the peel test was obtained by the AFM technique. This technique investigated the adhesion mechanism between LDPE and Al in LDPE/Al laminates. The AFM images of the LDPE and Al surfaces before and after peel tests are shown in Figure 6. The surface of unmodified LDPE excelled with a relatively smooth surface morphology/topography with a characteristic texture originating from the production process, while the Ra value was 3.6 nm. Modifying LDPE with 0.1 wt.% of maleated PE additive did not lead to significant changes in surface roughness (Ra = 4.0 nm), indicating incorporating additive mainly into the bulk of the LDPE matrix. However, significant surface morphology/topography changes were observed after peel tests of the LDPE-maleated PE/Al laminate. The surface roughness of LDPE modified with maleated PE (Ra = 19.5 nm) significantly increased after peel tests due to the mechanical coupling/interlocking mechanism of adhesion. This is probably caused when melted LDPE penetrates the pores or cavities of the other material (Al) to be bonded together, resulting in the creation of adhesive bond strength (mechanical interlocking) [34]. Mechanical interlocking also represents a surface characteristic dealing with adhesion forces at the microscopic/macroscopic levels and not molecular levels [54]. Moreover, a significant increase in surface roughness of Al after peel tests was observed, while Ra values increased from 7.3 nm to 49.2 nm as a result of attached maleated PE-containing domains to the Al surface. These findings proved a remarkable mechanical interlocking mechanism of adhesion in the LDPE-maleated PE/Al laminate.

## 4. Conclusions

The adhesion of LDPE packaging grade to Al was improved successfully in this study by bulk modification of LDPE with polar additives. Commercially available additives based on ethylene copolymers with methacrylic acids or maleic anhydride grafted PE (maleated) containing polar functional groups were used for this modification. This modification improved LDPE’s adhesion characteristics due to LDPE’s enhanced wettability, which was proved by contact angle measurements. The best adhesion of LDPE/Al adhesive laminate was obtained for LDPE modified by maleated PE additive, while 0.1 wt.% additive content led to a significant increase in peel resistance (from zero to 105 N/m). This modification did not significantly change the surface morphology/topography as the additive was homogeneously mixed with LDPE in bulk. An additional increase of maleated PE content (0.5 wt.%) in LDPE led to strong adhesion in the LDPE/Al laminate, which was more than the cohesion strength in Al foil. Moreover, no significant changes in mechanical properties of the LDPE modified by maleated PE additive were observed. Based on these results, it can be concluded that adding 0.5 wt.% of maleated PE into LDPE improved the adhesion of LDPE/Al laminates satisfactorily, which can be utilized in various adhesion-required applications, such as TetraPak packaging.

## Figures and Tables

**Table 1:** The physical properties and potential uses of the tested LDPE grades.

Polymer Properties	EC02
Density @ 23 °C (ASTM D-1505)	0.923 g/cm^3^
Melt flow index 190 °C/2.16 kg (ASTM D-1238)	4.0 g/10 min
Melting Point (ASTM E-794)	108 °C
Recommended uses	Extrusion of very high-clarity blown and cast films

**Table 2:** Mechanical properties of the LDPE samples.

Sample	Tensile Strength (MPa)	Young’s Modulus (MPa)
Unmodified	12.2 ± 0.9	288.6 ± 18.6
Modified with 0.1 wt.% OREVAC	11.3 ± 1.4	269.5 ± 21.5
Modified with 0.5 wt.% OREVAC	10.6 ± 0.5	253.0 ± 7.5

## Data Availability

Not applicable.
